# Glycated Albumin *versus* Glycated Hemoglobin as a Glycemic Indicator in Diabetic Patients on Peritoneal Dialysis

**DOI:** 10.3390/ijms17050619

**Published:** 2016-04-25

**Authors:** Hiroki Kobayashi, Masanori Abe, Yoshinori Yoshida, Hiroko Suzuki, Noriaki Maruyama, Kazuyoshi Okada

**Affiliations:** Division of Nephrology, Hypertension and Endocrinology, Department of Internal Medicine, Nihon University School of Medicine, Tokyo 173-8610, Japan; kobayashihiroki2@gmail.com (H.K.); yosi5226@med.nihon-u.ac.jp (Y.Y.); fab@fb4.so-net.ne.jp (H.S.); maruyama.noriaki@nihon-u.ac.jp (N.M.); okada.kazuyoshi@nihon-u.ac.jp (K.O.)

**Keywords:** diabetes mellitus, glycated albumin, glycated hemoglobin, hemodialysis, peritoneal dialysis

## Abstract

Compared with glycated hemoglobin (HbA1c), glycated albumin (GA) is superior in estimating glycemic control in diabetic patients on hemodialysis (HD). However, the better index for assessment of glycemic control in diabetic patients on peritoneal dialysis (PD) and the impact of protein loss on GA are unknown. Twenty diabetic patients on HD were matched by age, sex, and baseline postprandial plasma glucose (PG) levels to 20 PD patients. PG, HbA1c, GA, and serum albumin levels were measured for six months. Protein loss in PD patients was estimated by measuring the protein concentration in the peritoneal dialysate and by 24 h urine collection. Although PG and HbA1c did not differ significantly between the groups, the PD group had significantly lower GA (17.8% *versus* 20.8%, *p* < 0.001) and GA/HbA1c ratio (2.95% *versus* 3.45%, *p* < 0.0001) than the HD group. Although the PG level correlated significantly with the GA levels in both groups, it was not correlated with the HbA1c levels in both groups. HbA1c level was negatively associated with erythropoiesis-stimulating agent (ESA) dose in both groups, whereas GA was not significantly associated with serum albumin, hemoglobin concentration, ESA dose, and protein loss. Multiple regression analysis identified GA as the only independent factor associated with PG in PD patients. Our results suggested that GA was not significantly associated with protein loss, hemoglobin, serum albumin, and ESA dose. Although GA might underestimate glycemic status, it provided a significantly better measure for estimating glycemic control than HbA1c, even in PD patients.

## 1. Introduction

Although appropriate control of blood pressure, strict control of plasma glucose (PG) concentration, and the use of an angiotensin-converting enzyme inhibitor may prevent the development and progression of diabetic nephropathy, many patients still progress to end-stage kidney disease. In actuality, diabetes mellitus is the most common cause of initiation of dialysis, accounting for nearly 45% of cases in the United States and about 43.8% in Japan [1,2]. In addition, diabetes causes neuropathy, retinopathy, and atherosclerosis, leading to cardiovascular events. Strict glycemic control has been demonstrated to have beneficial effects on the prognosis of diabetic patients with chronic kidney disease on hemodialysis (HD) [3,4,5]. For this reason, control of blood glucose levels in diabetic patients who are undergoing dialysis is important in order to reduce complications and the mortality rate.

Several markers are useful for measuring long-term blood glucose control; these include glycated albumin (GA), glycated hemoglobin (HbA1c), and 1,5-anhydroglucitol (1,5AG). HbA1c often underestimates glycemic control of HD patients because of reduced red blood cell survival or the use erythropoiesis-stimulating agent (ESA) [6,7,8]; measurement of 1,5AG is not feasible for dialysis patients because of urine loss. On the other hand, measurement of GA level has been demonstrated by some reports to be superior in estimating glycemic control in HD patients with diabetes [9,10]. However, in PD patients, proteinuria and albumin loss into the PD fluid may affect the GA level because of reduced exposure of serum albumin to glucose [11]. The use of the GA level as an indicator of glycemic control is not well understood.

This study was designed to assess the correlation between PG and GA in PD patients with diabetes compared with HD patients with diabetes. In addition, we aimed to evaluate whether GA is better than HbA1c as an indicator of glycemic control in PD patients with diabetes. Furthermore, the relationship between protein loss and GA in PD patients with diabetes was also explored.

## 2. Results

**Figure 1:**
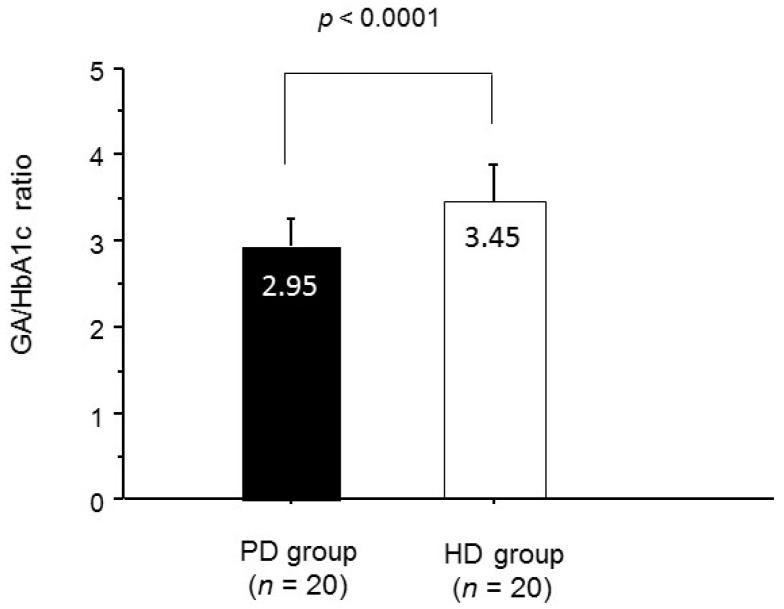
Comparison of glycated albumin (GA)/HbA1c ratio between the two groups.

As listed in Table 1, there was no significant difference in baseline characteristics between the two groups. None of the patients had changed their dose of anti-diabetic medications, and none in the PD group had changes in their PD prescriptions during the six month study period. Insulin was administered by subcutaneous injection to all patients who were treated with insulin. No significant differences were found in hemoglobin, ESA dose, and serum albumin levels between the two groups at baseline. Although PG and HbA1c did not differ significantly between the groups, GA was significantly lower in the PD group than in the HD group (17.8% *versus* 20.8%; *p* < 0.001). As shown in Figure 1, the GA/HbA1c ratio was significantly lower in the PD group (*p* < 0.0001).

**Figure 2:**
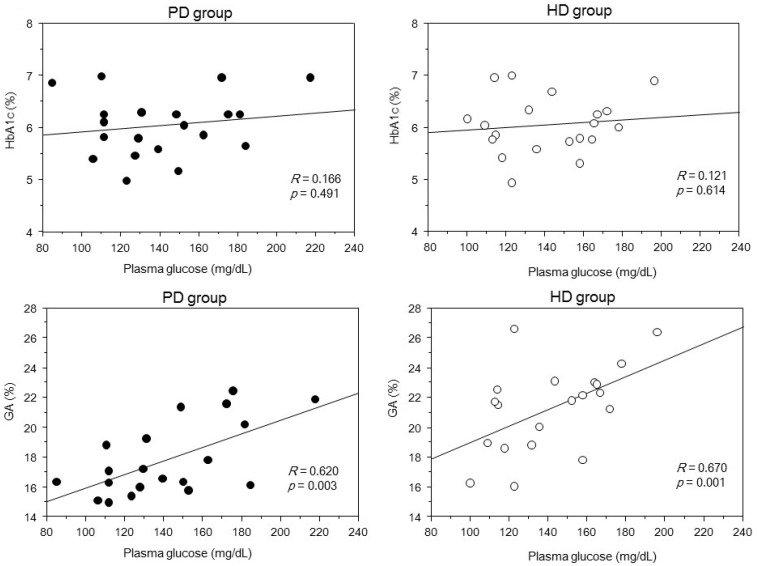
Association of mean plasma glucose level with mean HbA1c level and mean GA level in diabetic patients on dialysis. HD, hemodialysis; PD, peritoneal dialysis.

**Figure 3:**
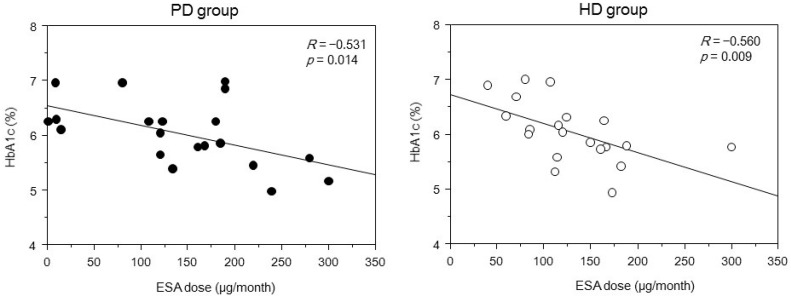
Relationship between mean HbA1c level and mean dose of monthly erythropoiesis-stimulating agent (ESA) in diabetic patients on dialysis. HD, hemodialysis; PD, peritoneal dialysis.

**Figure 4:**
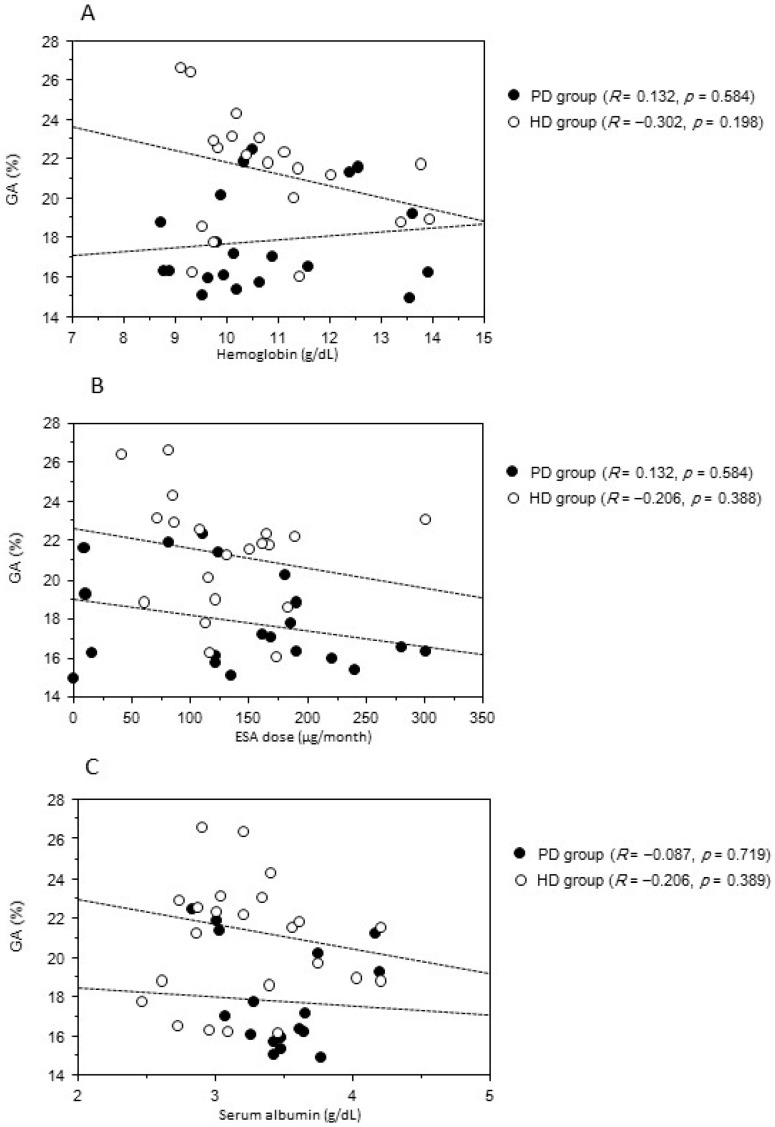

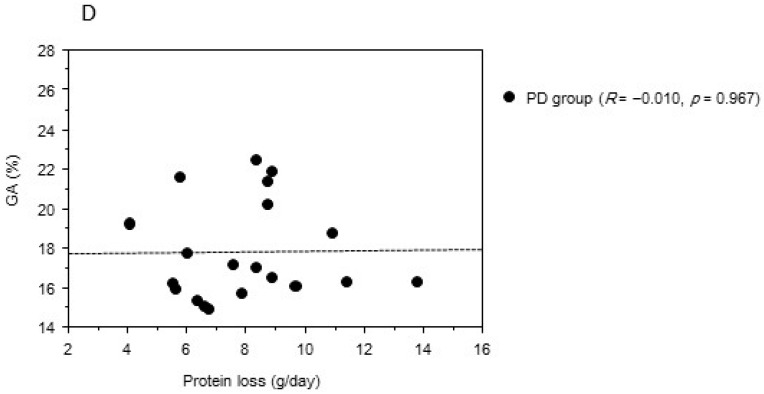
Associations of mean GA level with mean hemoglobin concentration (**A**); mean monthly ESA dosage (**B**); serum albumin level (**C**); and protein loss (**D**) in diabetic patients on dialysis. HD, hemodialysis; PD, peritoneal dialysis.

Figure 2 displays the correlations between mean HbA1c and GA levels with mean postprandial PG levels during six months. Although the PG level was not correlated with the HbA1c level, it correlated significantly with the GA level in both groups. Figure 3 depicts the relationship between HbA1c and ESA dose. ESA dose and HbA1c were negatively associated in both groups, while there was no significant association between HbA1c level and hemoglobin concentration in both groups. HbA1c level was not significantly associated with serum albumin level. Figure 4 shows that the GA level was not significantly associated with serum albumin, hemoglobin concentration, and dose of ESA in both groups. Furthermore, the GA level in the PD group was not associated with daily cumulative protein loss in the urine and PD fluid. In the multivariate analysis, GA was the only independent factor associated with PG in the two groups (Table 2).

## 3. Discussion

Several studies demonstrated that HbA1c tended to be lower in patients on HD compared with those who have residual kidney function; this is because of the elevation of immature erythrocytes due to blood loss during HD and ESA use for renal anemia [12]. Therefore, we often underestimate glycemic control in HD patients. On the other hand, serum GA was hypothesized to be an alternative marker for glycemic control in patients with type 2 diabetes because it is not affected by changes in erythrocyte survival time [7,8]. However, the usefulness of HbA1c and GA for PD patients is not well understood. The present study addressed two clinical issues. First, HbA1c assay had limitations as an indicator of glycemic control in diabetic patients who are undergoing PD. Second, GA was a better glycemic indicator than HbA1c in both HD and PD patients with diabetes.

In this study, hemoglobin concentrations were not associated with HbA1c levels, whereas there was a significant negative correlation between ESA dose and HbA1c level in both PD and HD patients. HbA1c level, which shows the percentage of glycated hemoglobin, reflects the concentration of serum glucose levels within 120 days before the test [13,14]; glycemic control a few weeks before the test could largely affect the HbA1c level. In HD patients, factors such as renal anemia caused by reduced erythrocyte life span due to uremia and metabolic acidosis may affect the accuracy of the HbA1c assay [6,7,8]. In addition, although PG levels remain constant, the use of ESAs might decrease HbA1c levels because ESA stimulates the production of erythrocytes and increases the peripheral blood proportion of immature erythrocytes, which were said to have lower glycated rates than mature erythrocytes [6,7,8]. Therefore, our study suggested that, regardless of dialysis type (HD or PD), HbA1c might be underestimated in patients who were treated with ESA.

The second clinical issue was that GA was a better glycemic indicator than HbA1c even in PD patients with diabetes. In this study, ESA dose, serum albumin, hemoglobin concentration, and the amount of protein loss in the PD group did not correlate with the GA level. Compared with HD patients, PD patients were reported to have longer red blood cell survival and shorter effect of ESA because of lower ESA requirements [15]. However, there was no difference in ESA dose among our study population. Therefore, the use of ESA might be a strong factor that led to reduction of HbA1c levels. It has been reported that GA showed a trend toward significance in HD (20.6%) *versus* PD (19.0%) patients and that the GA/HbA1c ratio differed between PD (2.77) and HD (3.02) patients, as well [16]. Those findings suggest the possibility that dialysate protein losses could impact GA in PD patients. Our study also showed that the GA/HbA1c ratio was higher in patients on HD than in patients on PD, regardless of similar PG levels for six months. This suggested that in patients on PD, GA measurements might significantly underestimate blood glucose levels compared with patients on HD. In patients who are undergoing PD, protein losses in the urine and PD fluid can significantly cause hypoalbuminemia; as such, we can hypothesize that GA levels can be underestimated because of the shortened exposure time of serum albumin to glucose in plasma. However, in the present study, protein loss and serum albumin level did not contribute to the GA level; in this situation, serum albumin levels were not decreased because albumin synthesis in the liver could make up for its loss in the urine and PD fluid. We also demonstrated that GA was the only independent factor affecting the PG level in the PD group. Therefore, our results suggested that GA may be a significantly better measure of glycemic control than HbA1c, in spite of the fact that GA might underestimate glycemic control in PD patients. Further studies would be needed to identify the factors affecting decreases in GA levels in PD patients.

Our study design was limited by the small sample size. Additional long-term studies are needed to accurately assess the effectiveness of GA in dialysis patients. In addition, we should also consider the differences in ethnicity, dosages of ESAs, HD flow rate, and body size. In the Dialysis Outcomes and Practice Patterns study involving 12 countries, Japanese HD patients received the lowest dosage of ESAs. On the other hand, HD patients in the United States received the highest dosage, which was over three times of dosage in Japan [17]. Despite the lower risk of underestimation of glycemic control by HbA1c compared with the US population, HbA1c was underestimated even in Japanese PD patients. Furthermore, the value of HbA1c may be affected in individuals living in countries where the prevalence of sickle hemoglobin (HbS) is high. In populations that commonly have the genetic variant HbS and, in cases wherein HbA1c determination has limited utility, physicians should select alternative methods of glycemic control determination, such as GA [18,19]. In addition, the differences in methods of albumin measurement should be taken into account when interpreting results from different countries. In this study, we used the new bromocresol purple method.

In conclusion, GA was a better glycemic indicator than HbA1c in PD patients with diabetes. The HbA1c assay had limitations as an indicator of glycemic control in diabetic patients on PD. In the future, further studies are needed to determine the target GA level that is necessary to ensure a good prognosis for diabetic patients on dialysis. Similarly, more data are needed to determine the stage of chronic kidney disease when GA levels are preferable over HbA1c levels for the assessment of glycemic control.

## 4. Experimental Section

### 4.1. Subjects and Study Design

This prospective, parallel-group, observational study was conducted between June 2014 and October 2015. The observation period was six months. PD patients eligible to participate in this study met the following criteria: (1) age ≥20 years and ≤80 years; (2) dialysis duration of >6 months at enrollment; (3) type 2 diabetes mellitus; (4) no change in hypoglycemic treatment during the preceding three months; and (5) no peritonitis episode during the previous six months. Exclusion criteria were as follows: (1) history of severe heart failure, angina, myocardial infarction, or stroke within the past six months; (2) presence of infectious disease, thyroid disease, malignant tumors, liver cirrhosis, hemolytic anemia, or treatment with steroids or immunosuppressants; and (3) current hospitalization. The eligible PD patients were matched with the same number of HD patients by age, sex, and baseline PG levels. This study included 20 PD patients who were matched with 20 HD patients. Patients were withdrawn from the study if they were started on combination PD and HD therapy, received blood transfusion, or had bleeding complications. The patients continued their regular medications, such as anti-diabetes drugs, anti-hypertensive drugs, ESA, phosphate binders, and lipid-lowering agents, during the study period. All patients received the same ESA (darbepoetin α).

The study protocol was approved by the ethics committee of our hospital and all patients provided written informed consent. The study protocol was designed in accordance with the Declaration of Helsinki.

### 4.2. Peritoneal Dialysis

Icodextrin solution and/or solutions containing glucose in the range of 1.35% to 2.5% were used as PD fluids. The PD fluids did not change during the observation period.

### 4.3. Hemodialysis

In all HD patients, HD was performed for 4 h at a blood flow rate of 200 mL/min and a dialysate flow rate of 500 mL/min. HD was performed using dialyzers containing high-flux membranes. The surface area of the dialyzer membrane was selected according to the patient’s body weight. The glucose concentration of the dialysate was 100 mg/dL. Heparin was administered for anticoagulation at a dose of 2600–5000 units per HD session. The ultrafiltration volume was maintained based on the clinical dry weight during each session.

### 4.4. Study Evaluations

The levels of PG, HbA1c, GA, and serum albumin were measured monthly. In PD patients, protein loss was estimated by measuring the protein concentration in the PD effluents and the total effluent volume obtained from the 24 h urine collection. Postprandial PG levels were measured in both groups. In patients with HD, blood samples were obtained before the start of HD session. GA was measured by an enzymatic method using the Lucica GA-L^®^ Kit (Asahi Kasei Pharma Co., Tokyo, Japan). The protein concentrations in the PD effluents and urine were measured by the pyrogallol red method. A fast peritoneal equilibration test (PET) was performed in all PD patients to evaluate peritoneal transport characteristics at baseline.

### 4.5. Statistical Analyses

Data were expressed as mean ± standard deviation or median (interquartile range), as appropriate. Continuous variables were compared using Student’s *t*-test or Mann–Whitney *U* test, as appropriate; categorical variables were compared using the χ^2^ test or Fisher’s exact test, as appropriate. Multivariate regression analysis with mean PG level as the dependent variable and mean HbA1c, GA, hemoglobin, serum albumin level, ESA dose, and protein loss as the independent variables was performed to investigate the predictor of mean PG levels in each group. Statistical significance was set at *p* < 0.05. All analyses were performed using JMP software version 12 (SAS Institute Ltd., Cary, NC, USA).

## Figures and Tables

**Table 1:** Comparison of diabetic patients based on type of dialysis received.

Variable	PD Group	HD Group	*p* Value
Number of the patients	20	20	‒
Gender (male %)	85	80	0.687
Age (years)	59.6 ± 9.5	58.6 ± 7.4	0.712
Duration of dialysis (m)	23.6 ± 18.3	24.8 ± 11.8	0.798
Body mass index (kg/m^2^)	24.3 ± 2.9	23.9 ± 1.8	0.704
Hemoglobin (g/dL)	10.8 ± 1.6	10.8 ± 1.5	0.876
Serum albumin (g/dL)	3.4 ± 0.4	3.5 ± 0.4	0.404
Plasma glucose (mg/dL)	142 ± 33	142 ± 27	0.996
Glycated hemoglobin (%)	6.0 ± 0.6	6.1 ± 0.7	0.951
Glycated albumin (%)	17.8 ± 2.5	20.8 ± 2.8	<0.001
Darbepoetin dose (μg/month)	143 ± 89	130 ± 58	0.596
Antidiabetes therapy (*n* (%))			0.836
Diet modification alone	4 (20)	4 (25)	
DPP-4 inhibitors	7 (35)	9 (45)	
Repaglinide	3 (15)	3 (15)	
α-GIs	1 (5)	2 (10)	
Insulin	8 (40)	7 (35)	
PET (*n*)			
H/HA/LA/L	2/8/8/2	‒	‒
Protein loss (g/day)	8.0 ± 2.3	‒	‒

DPP-4, dipeptidyl peptidase-IV; GI, glucosidase inhibitor; H, high; HA, high average; HD, hemodialysis; L, low; LA, low average; PD, peritoneal dialysis; PET, peritoneal equilibration test.

**Table 2:** Multivariate analysis of clinical factors affecting plasma glucose level in diabetic patients on dialysis.

	PD Group (*R*^2^ = 0.51)					HD Group (*R*^2^ = 0.63)				
Variable	β	SE	95% CI		*p* Value	β	SE	95% CI		*p* Value
			Lower	Upper				Lower	Upper	
HbA1c	−30.8	21.1	−76.6	14.9	0.168	−15.6	10.2	−37.6	6.2	0.147
GA	11.8	2.7	3.8	19.7	0.007	7.2	1.7	3.6	10.9	0.0008
Hemoglobin	−5.6	6.3	−19.2	8.1	0.389	1.9	4.7	−8.1	12.1	0.688
Serum albumin	−3.2	20.1	−46.6	40.3	0.877	−26.6	14.5	−57.7	4.4	0.086
ESA dose	−0.2	0.2	−0.5	0.2	0.341	−0.08	0.1	−0.3	0.1	0.386
Protein loss	1.2	4.7	−9.1	11.4	0.801					

ESA, erythropoiesis-stimulating agent; GA, glycated albumin; HbA1c, glycated hemoglobin; HD, hemodialysis; PD, peritoneal dialysis; SE, standard error.
